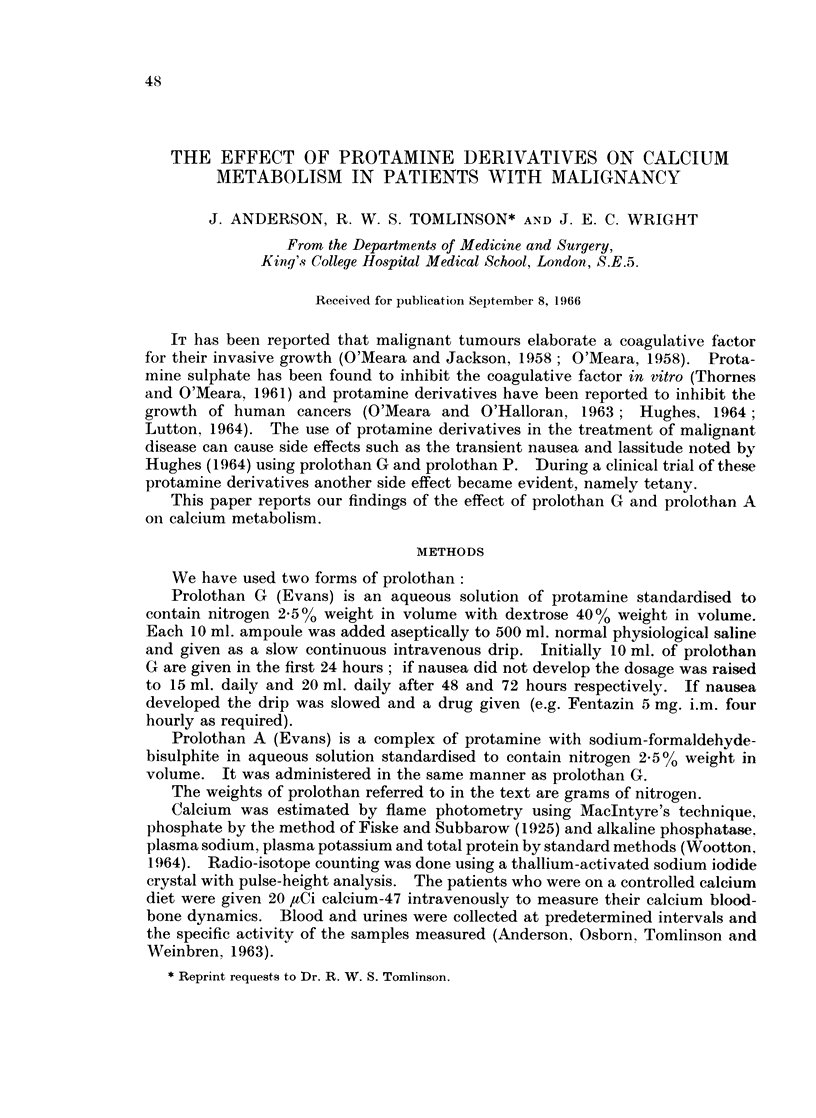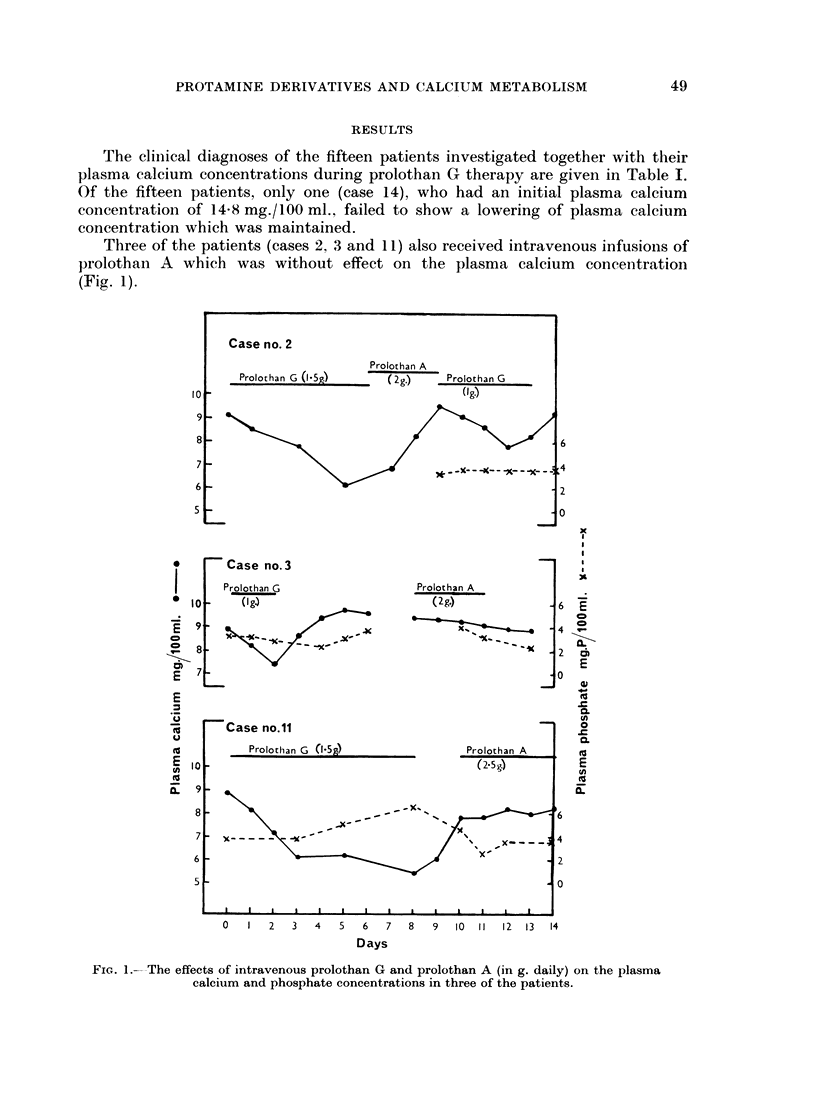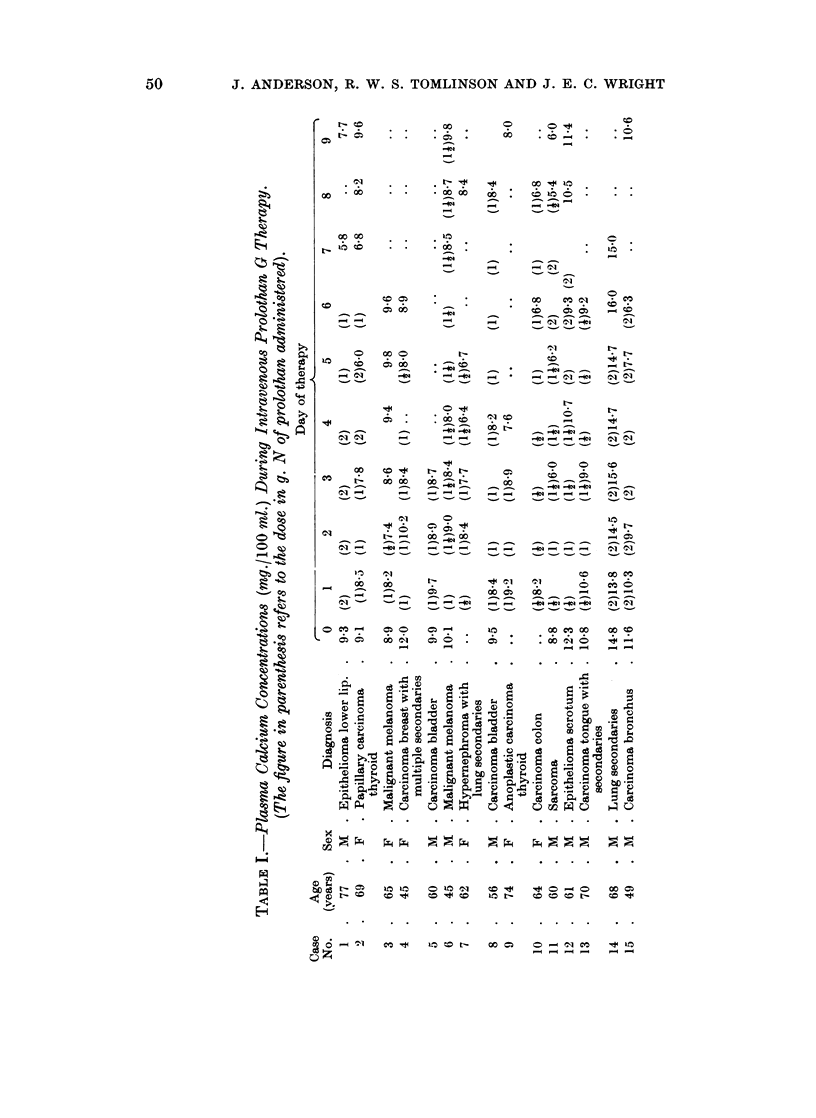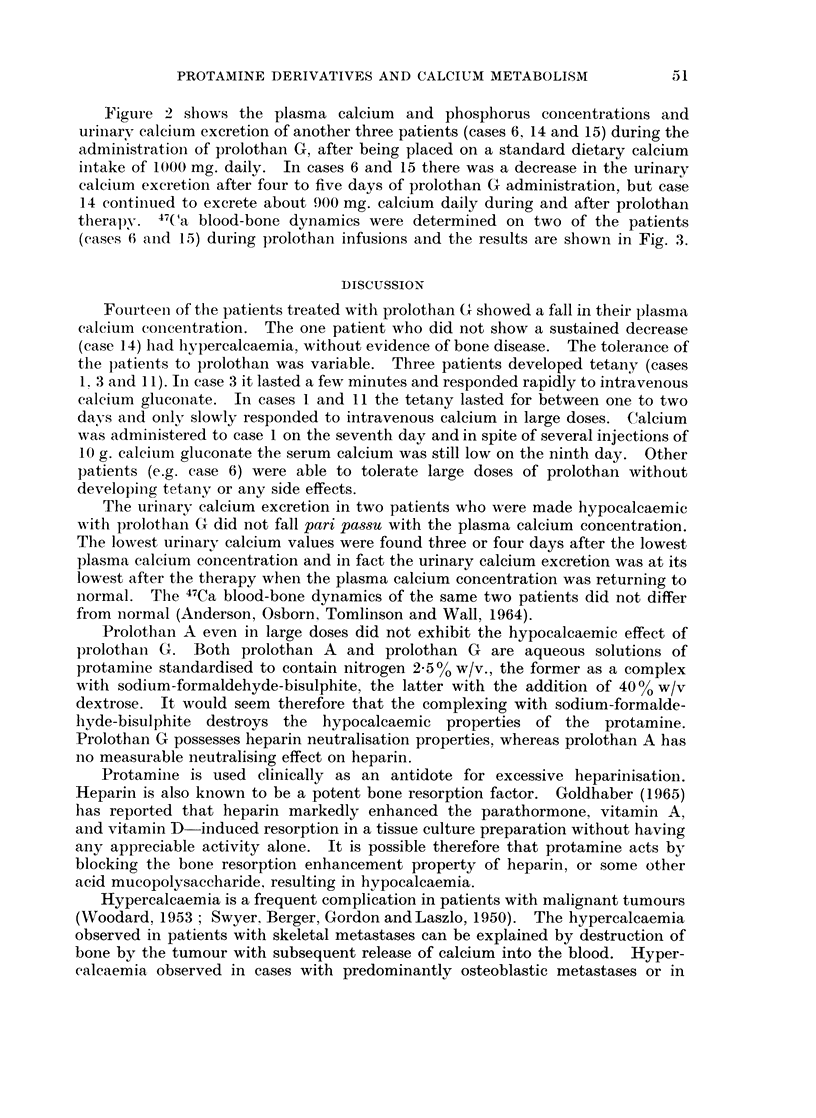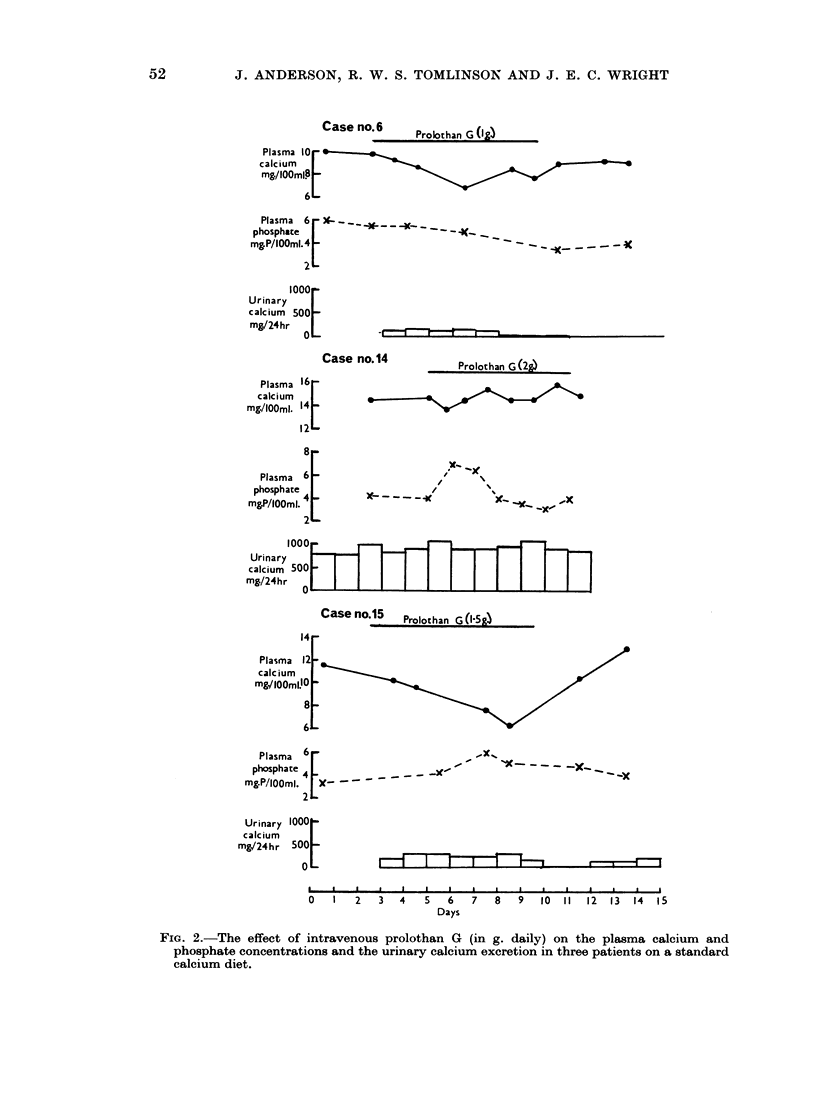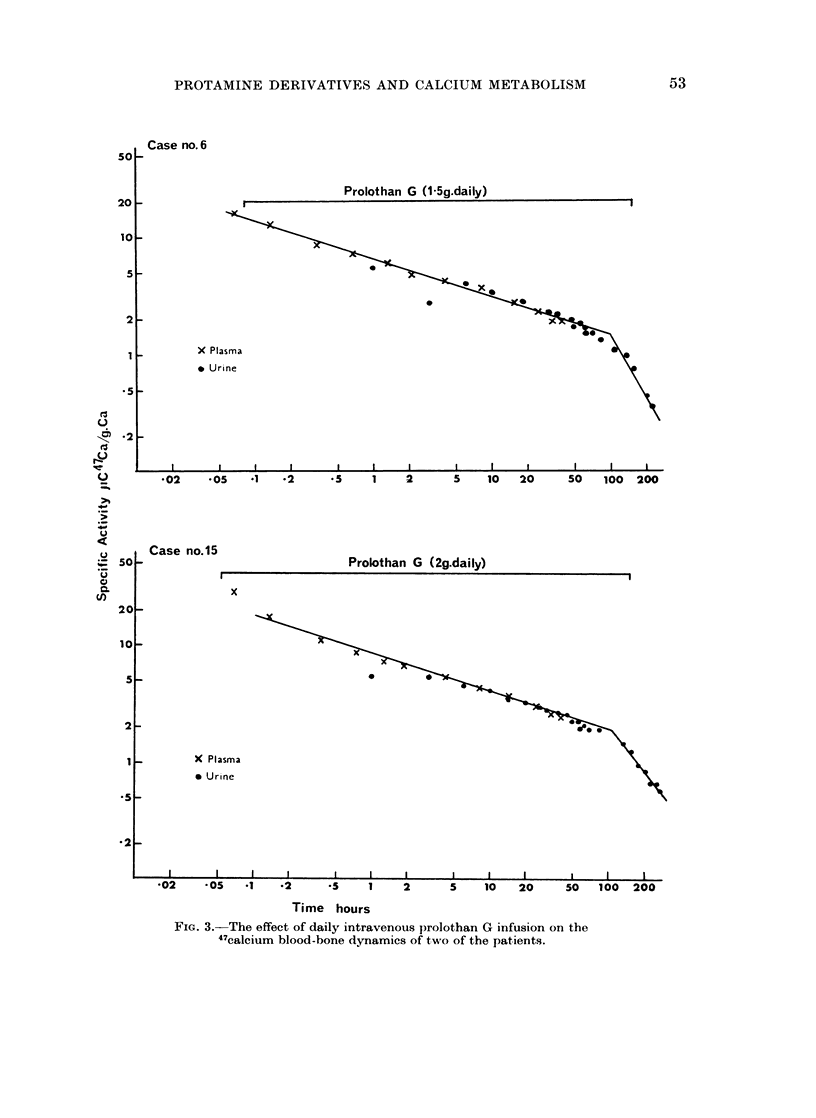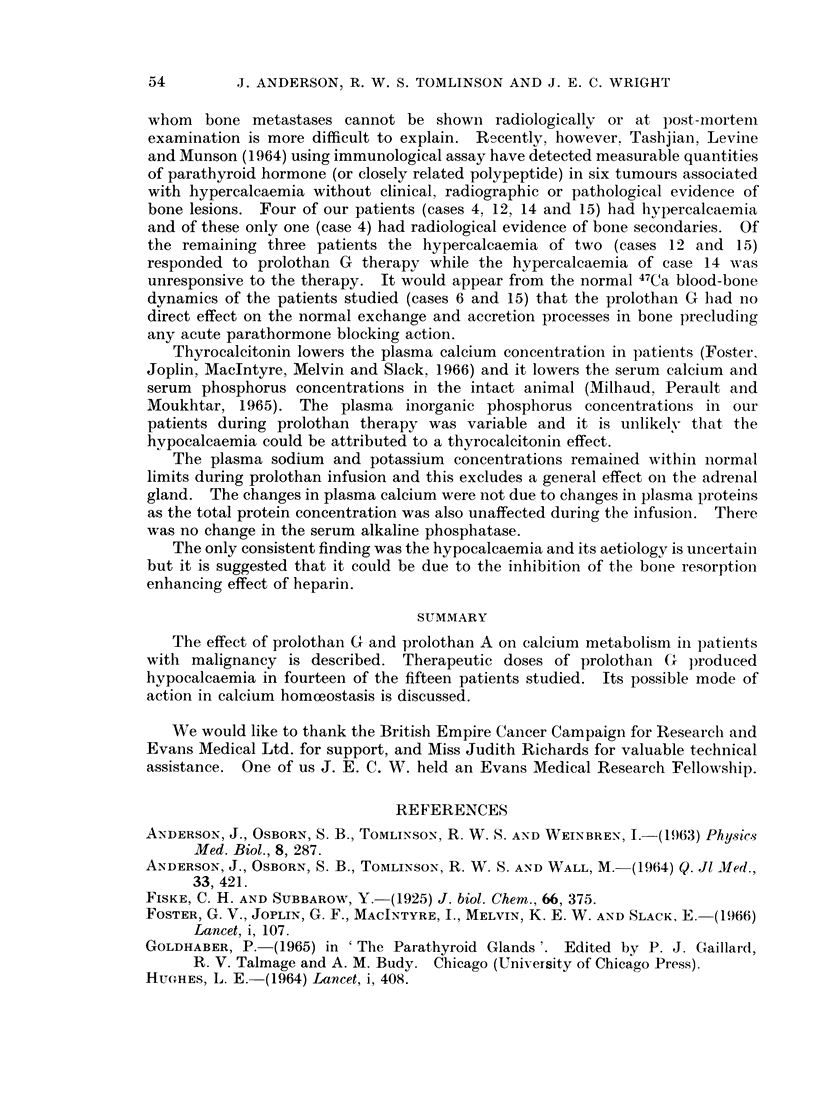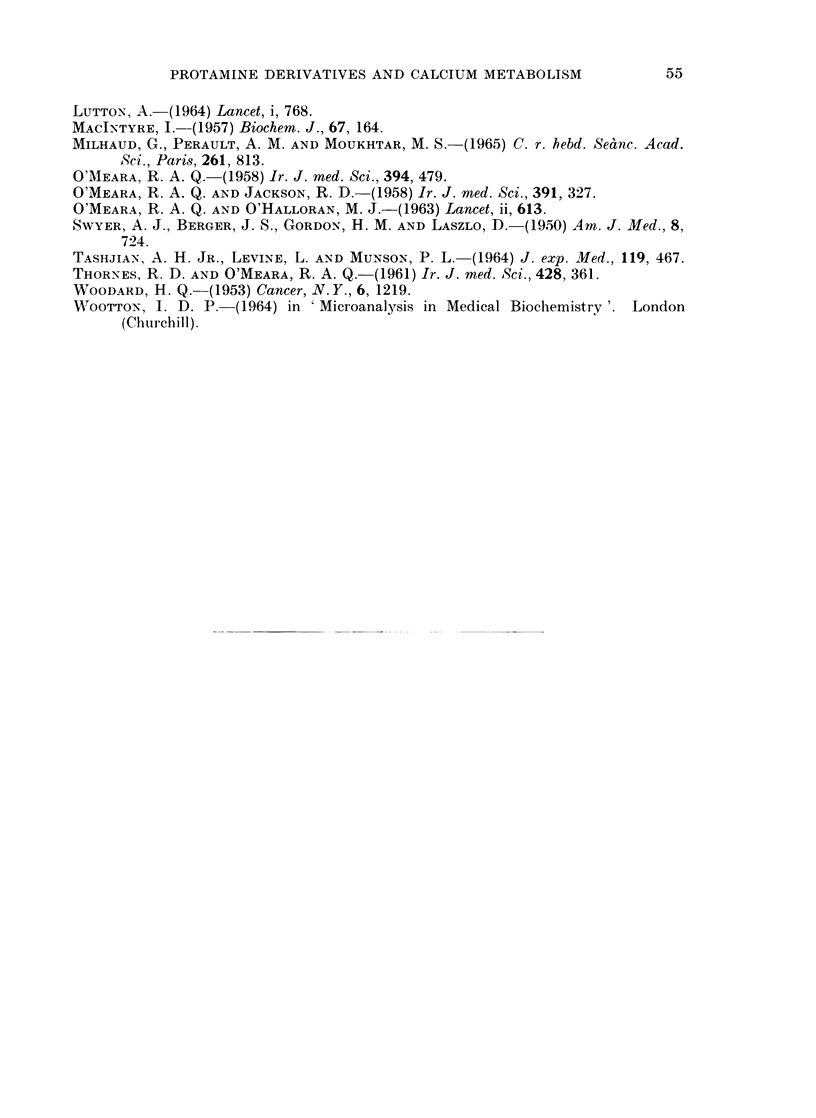# The effect of protamine derivatives on calcium metabolism in patients with malignancy.

**DOI:** 10.1038/bjc.1967.7

**Published:** 1967-03

**Authors:** J. Anderson, R. W. Tomlinson, J. E. Wright


### THE EFFECT OF PROTAMINE DERIVATIVES ON CALCIUM METABOLISM IN PATIENTS WITH MALIGNANCY

J. ANDERSON, R. W. S. TOMLINSON* AND J. E. C. WRIGHT

*From the Departments of Medicine and Surgery, King's College Hospital Medical School, London, S.E.5.*

Received for publication September 8, 1966

It has been reported that malignant tumours elaborate a coagulative factor for their invasive growth (O'Meara and Jackson, 1958 ; O'Meara, 1958). Protamine sulphate has been found to inhibit the coagulative factor *in vitro* (Thornes and O'Meara, 1961) and protamine derivatives have been reported to inhibit the growth of human cancers (O'Meara and O'Halloran, 1963 ; Hughes, 1964 ; Lutton, 1964). The use of protamine derivatives in the treatment of malignant disease can cause side effects such as the transient nausea and lassitude noted by Hughes (1964) using prolothan G and prolothan P. During a clinical trial of these protamine derivatives another side effect became evident, namely tetany.

This paper reports our findings of the effect of prolothan G and prolothan A on calcium metabolism.

### METHODS

We have used two forms of prolothan :

Prolothan G (Evans) is an aqueous solution of protamine standardised to contain nitrogen 2·5% weight in volume with dextrose 40% weight in volume. Each 10 ml. ampoule was added aseptically to 500 ml. normal physiological saline and given as a slow continuous intravenous drip. Initially 10 ml. of prolothan G are given in the first 24 hours ; if nausea did not develop the dosage was raised to 15 ml. daily and 20 ml. daily after 48 and 72 hours respectively. If nausea developed the drip was slowed and a drug given (e.g. Fentazin 5 mg. i.m. four hourly as required).

Prolothan A (Evans) is a complex of protamine with sodium-formaldehyde-bisulphite in aqueous solution standardised to contain nitrogen 2·5% weight in volume. It was administered in the same manner as prolothan G.

The weights of prolothan referred to in the text are grams of nitrogen.

Calcium was estimated by flame photometry using MacIntyre's technique, phosphate by the method of Fiske and Subbarow (1925) and alkaline phosphatase, plasma sodium, plasma potassium and total protein by standard methods (Wootton, 1964). Radio-isotope counting was done using a thallium-activated sodium iodide crystal with pulse-height analysis. The patients who were on a controlled calcium diet were given 20 $\mu$Ci calcium-47 intravenously to measure their calcium blood-bone dynamics. Blood and urines were collected at predetermined intervals and the specific activity of the samples measured (Anderson, Osborn, Tomlinson and Weinbren, 1963).

* Reprint requests to Dr. R. W. S. Tomlinson.

### RESULTS

The clinical diagnoses of the fifteen patients investigated together with their plasma calcium concentrations during prolothan G therapy are given in Table I. Of the fifteen patients, only one (case 14), who had an initial plasma calcium concentration of 14·8 mg./100 ml., failed to show a lowering of plasma calcium concentration which was maintained.

Three of the patients (cases 2, 3 and 11) also received intravenous infusions of prolothan A which was without effect on the plasma calcium concentration (Fig. 1).

FIG. 1.—The effects of intravenous prolothan G and prolothan A (in g. daily) on the plasma calcium and phosphate concentrations in three of the patients.

TABLE I.—*Plasma Calcium Concentrations (mg./100 ml.) During Intravenous Prolothan G Therapy. (The figure in parenthesis refers to the dose in g. N of prolothan administered).*

| Case No. | Age (years) | Sex | Diagnosis | Day of therapy 0 | 1 | 2 | 3 | 4 | 5 | 6 | 7 | 8 | 9 |
|---|---|---|---|---|---|---|---|---|---|---|---|---|---|
| 1 | 77 | M | Epithelioma lower lip. | 9·3 | (2) | (2) | (2) | (2) | (1) | (1) | 5·8 | .. | 7·7 |
| 2 | 69 | F | Papillary carcinoma thyroid | 9·1 | (1)8·5 | (1) | (1)7·8 | (2) | (2)6·0 | (1) | 6·8 | 8·2 | 9·6 |
| 3 | 65 | F | Malignant melanoma | 8·9 | (1)8·2 | (½)7·4 | 8·6 | 9·4 | 9·8 | 9·6 | .. | .. | .. |
| 4 | 45 | F | Carcinoma breast with multiple secondaries | 12·0 | (1) | (1)10·2 | (1)8·4 | (1) .. | (½)8·0 | 8·9 | .. | .. | .. |
| 5 | 60 | M | Carcinoma bladder | 9·9 | (1)9·7 | (1)8·9 | (1)8·7 | .. | .. | .. | .. | .. | .. |
| 6 | 45 | M | Malignant melanoma | 10·1 | (1) | (1½)9·0 | (1½)8·4 | (1½)8·0 | (1½) | (1½) | (1½)8·5 | (1½)8·7 | (1½)9·8 |
| 7 | 62 | F | Hypernephroma with lung secondaries | .. | (½) | (1)8·4 | (1)7·7 | (1½)6·4 | (½)6·7 | .. | .. | 8·4 | .. |
| 8 | 56 | M | Carcinoma bladder | 9·5 | (1)8·4 | (1) | (1) | (1)8·2 | (1) | (1) | (1) | (1)8·4 | |
| 9 | 74 | F | Anoplastic carcinoma thyroid | .. | (1)9·2 | (1) | (1)8·9 | 7·6 | .. | .. | .. | .. | 8·0 |
| 10 | 64 | F | Carcinoma colon | .. | (½)8·2 | (½) | (½) | (½) | (1) | (1)6·8 | (1) | (1)6·8 | .. |
| 11 | 60 | M | Sarcoma | 8·8 | (½) | (1) | (1½)6·0 | (1½) | (1½)6·2 | (2) | (2) | (½)5·4 | 6·0 |
| 12 | 61 | M | Epithelioma scrotum | 12·3 | (½) | (1) | (1½) | (1½)10·7 | (2) | (2)9·3 | (2) | 10·5 | 11·4 |
| 13 | 70 | M | Carcinoma tongue with secondaries | 10·8 | (½)10·6 | (1) | (1½)9·0 | (½) | (½) | (½)9·2 | .. | .. | .. |
| 14 | 68 | M | Lung secondaries | 14·8 | (2)13·8 | (2)14·5 | (2)15·6 | (2)14·7 | (2)14·7 | 16·0 | 15·0 | .. | .. |
| 15 | 49 | M | Carcinoma bronchus | 11·6 | (2)10·3 | (2)9·7 | (2) | (2) | (2)7·7 | (2)6·3 | .. | .. | 10·6 |

Figure 2 shows the plasma calcium and phosphorus concentrations and urinary calcium excretion of another three patients (cases 6, 14 and 15) during the administration of prolothan G, after being placed on a standard dietary calcium intake of 1000 mg. daily. In cases 6 and 15 there was a decrease in the urinary calcium excretion after four to five days of prolothan G administration, but case 14 continued to excrete about 900 mg. calcium daily during and after prolothan therapy. $^{47}Ca$ blood-bone dynamics were determined on two of the patients (cases 6 and 15) during prolothan infusions and the results are shown in Fig. 3.

### DISCUSSION

Fourteen of the patients treated with prolothan G showed a fall in their plasma calcium concentration. The one patient who did not show a sustained decrease (case 14) had hypercalcaemia, without evidence of bone disease. The tolerance of the patients to prolothan was variable. Three patients developed tetany (cases 1, 3 and 11). In case 3 it lasted a few minutes and responded rapidly to intravenous calcium gluconate. In cases 1 and 11 the tetany lasted for between one to two days and only slowly responded to intravenous calcium in large doses. Calcium was administered to case 1 on the seventh day and in spite of several injections of 10 g. calcium gluconate the serum calcium was still low on the ninth day. Other patients (e.g. case 6) were able to tolerate large doses of prolothan without developing tetany or any side effects.

The urinary calcium excretion in two patients who were made hypocalcaemic with prolothan G did not fall *pari passu* with the plasma calcium concentration. The lowest urinary calcium values were found three or four days after the lowest plasma calcium concentration and in fact the urinary calcium excretion was at its lowest after the therapy when the plasma calcium concentration was returning to normal. The $^{47}Ca$ blood-bone dynamics of the same two patients did not differ from normal (Anderson, Osborn, Tomlinson and Wall, 1964).

Prolothan A even in large doses did not exhibit the hypocalcaemic effect of prolothan G. Both prolothan A and prolothan G are aqueous solutions of protamine standardised to contain nitrogen 2·5% w/v., the former as a complex with sodium-formaldehyde-bisulphite, the latter with the addition of 40% w/v dextrose. It would seem therefore that the complexing with sodium-formaldehyde-bisulphite destroys the hypocalcaemic properties of the protamine. Prolothan G possesses heparin neutralisation properties, whereas prolothan A has no measurable neutralising effect on heparin.

Protamine is used clinically as an antidote for excessive heparinisation. Heparin is also known to be a potent bone resorption factor. Goldhaber (1965) has reported that heparin markedly enhanced the parathormone, vitamin A, and vitamin D—induced resorption in a tissue culture preparation without having any appreciable activity alone. It is possible therefore that protamine acts by blocking the bone resorption enhancement property of heparin, or some other acid mucopolysaccharide, resulting in hypocalcaemia.

Hypercalcaemia is a frequent complication in patients with malignant tumours (Woodard, 1953 ; Swyer, Berger, Gordon and Laszlo, 1950). The hypercalcaemia observed in patients with skeletal metastases can be explained by destruction of bone by the tumour with subsequent release of calcium into the blood. Hypercalcaemia observed in cases with predominantly osteoblastic metastases or in

FIG. 2.—The effect of intravenous prolothan G (in g. daily) on the plasma calcium and phosphate concentrations and the urinary calcium excretion in three patients on a standard calcium diet.

FIG. 3.—The effect of daily intravenous prolothan G infusion on the $^{47}$calcium blood-bone dynamics of two of the patients.

whom bone metastases cannot be shown radiologically or at post-mortem examination is more difficult to explain. Recently, however, Tashjian, Levine and Munson (1964) using immunological assay have detected measurable quantities of parathyroid hormone (or closely related polypeptide) in six tumours associated with hypercalcaemia without clinical, radiographic or pathological evidence of bone lesions. Four of our patients (cases 4, 12, 14 and 15) had hypercalcaemia and of these only one (case 4) had radiological evidence of bone secondaries. Of the remaining three patients the hypercalcaemia of two (cases 12 and 15) responded to prolothan G therapy while the hypercalcaemia of case 14 was unresponsive to the therapy. It would appear from the normal $^{47}Ca$ blood-bone dynamics of the patients studied (cases 6 and 15) that the prolothan G had no direct effect on the normal exchange and accretion processes in bone precluding any acute parathormone blocking action.

Thyrocalcitonin lowers the plasma calcium concentration in patients (Foster, Joplin, MacIntyre, Melvin and Slack, 1966) and it lowers the serum calcium and serum phosphorus concentrations in the intact animal (Milhaud, Perault and Moukhtar, 1965). The plasma inorganic phosphorus concentrations in our patients during prolothan therapy was variable and it is unlikely that the hypocalcaemia could be attributed to a thyrocalcitonin effect.

The plasma sodium and potassium concentrations remained within normal limits during prolothan infusion and this excludes a general effect on the adrenal gland. The changes in plasma calcium were not due to changes in plasma proteins as the total protein concentration was also unaffected during the infusion. There was no change in the serum alkaline phosphatase.

The only consistent finding was the hypocalcaemia and its aetiology is uncertain but it is suggested that it could be due to the inhibition of the bone resorption enhancing effect of heparin.

### SUMMARY

The effect of prolothan G and prolothan A on calcium metabolism in patients with malignancy is described. Therapeutic doses of prolothan G produced hypocalcaemia in fourteen of the fifteen patients studied. Its possible mode of action in calcium homœostasis is discussed.

We would like to thank the British Empire Cancer Campaign for Research and Evans Medical Ltd. for support, and Miss Judith Richards for valuable technical assistance. One of us J. E. C. W. held an Evans Medical Research Fellowship.

### REFERENCES

Anderson, J., Osborn, S. B., Tomlinson, R. W. S. and Weinbren, I.—(1963) *Physics Med. Biol.*, **8**, 287.

Anderson, J., Osborn, S. B., Tomlinson, R. W. S. and Wall, M.—(1964) *Q. Jl Med.*, **33**, 421.

Fiske, C. H. and Subbarow, Y.—(1925) *J. biol. Chem.*, **66**, 375.

Foster, G. V., Joplin, G. F., MacIntyre, I., Melvin, K. E. W. and Slack, E.—(1966) *Lancet*, i, 107.

Goldhaber, P.—(1965) in 'The Parathyroid Glands'. Edited by P. J. Gaillard, R. V. Talmage and A. M. Budy. Chicago (University of Chicago Press).

Hughes, L. E.—(1964) *Lancet*, i, 408.

LUTTON, A.—(1964) *Lancet*, i, 768.
MACINTYRE, I.—(1957) *Biochem. J.*, **67**, 164.
MILHAUD, G., PERAULT, A. M. AND MOUKHTAR, M. S.—(1965) *C. r. hebd. Seànc. Acad. Sci., Paris*, **261**, 813.
O'MEARA, R. A. Q.—(1958) *Ir. J. med. Sci.*, **394**, 479.
O'MEARA, R. A. Q. AND JACKSON, R. D.—(1958) *Ir. J. med. Sci.*, **391**, 327.
O'MEARA, R. A. Q. AND O'HALLORAN, M. J.—(1963) *Lancet*, ii, **613**.
SWYER, A. J., BERGER, J. S., GORDON, H. M. AND LASZLO, D.—(1950) *Am. J. Med.*, **8**, 724.
TASHJIAN, A. H. JR., LEVINE, L. AND MUNSON, P. L.—(1964) *J. exp. Med.*, **119**, 467.
THORNES, R. D. AND O'MEARA, R. A. Q.—(1961) *Ir. J. med. Sci.*, **428**, 361.
WOODARD, H. Q.—(1953) *Cancer, N.Y.*, **6**, 1219.
WOOTTON, I. D. P.—(1964) in 'Microanalysis in Medical Biochemistry'. London (Churchill).